# Parallel Pandemics Illustrate the Need for One Health Solutions

**DOI:** 10.3389/fmicb.2021.718546

**Published:** 2021-10-08

**Authors:** Claire Tucker, Anna Fagre, George Wittemyer, Tracy Webb, Edward Okoth Abworo, Sue VandeWoude

**Affiliations:** ^1^Department of Microbiology, Immunology, and Pathology, College of Veterinary Medicine and Biomedical Sciences, Colorado State University, Fort Collins, CO, United States; ^2^Department of Fish, Wildlife, and Conservation Biology, Warner College of Natural Resources, Colorado State University, Fort Collins, CO, United States; ^3^Department of Clinical Sciences, College of Veterinary Medicine and Biomedical Sciences, Colorado State University, Fort Collins, CO, United States; ^4^Department of Animal and Human Health, International Livestock Research Institute, Nairobi, Kenya

**Keywords:** African Swine Fever, COVID-19, One Health, disease surveillance and control, public health

## Abstract

African Swine Fever (ASF) was reported in domestic pigs in China in 2018. This highly contagious viral infection with no effective vaccine reached pandemic proportions by 2019, substantially impacting protein availability in the same region where the COVID-19 pandemic subsequently emerged. We discuss the genesis, spread, and wide-reaching impacts of this epidemic in a vital livestock species, noting parallels and potential contributions to ignition of COVID-19. We speculate about impacts of these pandemics on global public health infrastructure and suggest intervention strategies using a cost: benefit approach for low-risk, massive-impact events. We note that substantive changes in how the world reacts to potential threats will be required to overcome catastrophes driven by climate change, food insecurity, lack of surveillance infrastructure, and other gaps. A One Health approach creating collaborative processes connecting expertise in human, animal, and environmental health is essential for combating future global health crises.

## Introduction

The Centers for Disease Control and Prevention (CDC), the One Health Commission, the United States Department of Agriculture (USDA), and the National Institutes of Health (NIH) define One Health as an approach, involving health of humans, animals (domestic and wild), and the environment (ecosystem and sometimes plants), and involving a wide lens and transdisciplinary effort (Centers for Disease Control, 2020a). The One Health Initiative Task Force, convened by the American Veterinary Medical Association (AVMA), defines One Health succinctly as: “the collaborative efforts of multiple disciplines working locally, nationally, and globally, to attain optimal health for people, animals and our environment” (American Veterinary Medical Association, 2008).

The COVID-19 pandemic, resulting from transmission of the newly discovered SARS-CoV, is one of the most severe crises of the Anthropocene. Examining the reasons underlying the emergence of SARS-CoV-2, its epidemic spread, effective control measures, and unforeseen consequences of COVID-19 will occupy us for decades. Yet the COVID-19 pandemic did not arise in a vacuum. A second pandemic caused by African Swine Fever virus (ASFV) emerged in domestic swine populations in China just prior to COVID-19, spreading to Mongolia, Vietnam, and Eastern Europe by mid-2019. The ASF panzootic, while caused by a different virus in a different species, has strikingly parallel drivers to the COVID-19 pandemic, and impacts of both infections have multiplied far beyond the original threat, overwhelming government and agency capacity to monitor and mitigate their initial spread.

Here we describe the temporal and thematic links that reveal similar patterns in these two threats and discuss factors associated with ASF that compounded the COVID-19 pandemic. Commonalities between these pandemics include concerns surrounding transmission to and from wildlife, interconnected global networks, and concomitant stresses on food supply and disease surveillance capacity. Future consequences of these pandemics include exacerbation of food insecurity and bottlenecks in disease surveillance capacity. These two pandemics underscore the need to use a One Health framework, incorporating diverse experts cooperating globally, to overcome continuing threats.

## African Swine Fever

With Chinese citizens consuming 28% of the global meat production, China has emerged as the primary pork-producing country, with half of the world’s pigs – upward of 700 million head per year – being raised in China. While Chinese pork production has historically been managed by smaller farming units, modern intensive swine facilities have flourished over the last decade to meet growing demands (Cheng et al., 2011).

The Chinese pork market had been largely unhindered by serious disease during its expansion and intensification. However, production was decimated by the recent emergence of ASF, a viral infection endemic in domestic and wild suids that results in fever, gastrointestinal disease, and respiratory illness typically leading to death (Penrith and Vosloo, 2009; O’Neill et al., 2020). ASF has been associated with serious economic ramifications during outbreaks due to high mortality, the use of culling as for primary control, and trade restrictions with unaffected countries. Unlike diseases like classical swine fever and pseudorabies, ASF does not have an efficacious vaccine to control its impact.

The first case of ASF reported in the domestic pigs in China was in August 2018 (Wang et al., 2019). In order to halt the spread of the infection, the Chinese government mandated strict culling laws, with a recommendation to slaughter every pig within 3 km of a known infection (Staff, 2019). Despite these aggressive control measures, ASF spread to all mainland provinces. Estimates of the number of slaughtered pigs range from 150–200 million, which represents 30% of all Chinese pigs, though the true figure may approach 50–70% of the total pig population (Berthe, 2020; Good, 2021). Although the economic impacts of ASF are still being tallied, some scenarios have calculated a 1% reduction in China’s GDP ($100 billion USD) (Ma et al., 2020). It is also estimated that the incursion of ASF into China killed half of breeding sow stocks, resulting in lower production of pigs (China Ministry of Agriculture). The virus has additionally spread to many Asian countries including Vietnam, Cambodia, Indonesia, and India, causing significant impacts to pork production across Asia and Europe (Mazur-Panasiuk et al., 2019), and ASF was recently reported in several feral swine in Eastern Germany (Standaert, 2020a). ASF has not however, spread globally, because of strict trade restrictions with other countries and the aforementioned culling policies. However, there is a very real threat the United States pig industry could be affected by deliberate or accidental introduction of ASF virus.

**FIGURE 1:**
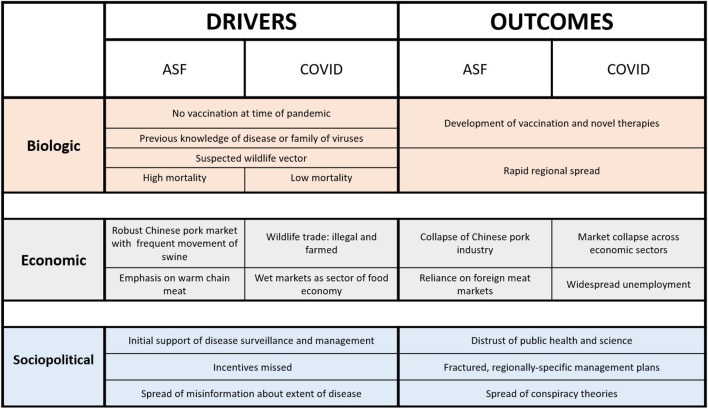
Drivers and outcomes of ASF and COVID have animal, human, and environmental health implications. This comparative framework identifies commonalities and predictable aspects of One Health pandemics.

The rapid spread of ASF has been influenced by many factors, some intrinsically related to the virus and others to governance, culture, and economy (Figure 1). The economic trend for consolidation of pork production in intensive rearing conditions increases risk of disease, with pig density identified as the most important predictor of an ASF outbreak (Ma et al., 2020). ASF is a hardy and stable virus, reported to survive high temperatures and freezing, and can live for days on food products, fomites, and other pigs (Mazur-Panasiuk et al., 2019). The feeding of kitchen waste (including both raw and cooked pork) is a common cultural practice in China, which results in a rapid chain of transmission between animals, if the waste is contaminated with ASF virus. Pig density was identified as the most important predictor of an ASF outbreak; thus, the economic drive trend for consolidation of pork production in intensive rearing conditions has also contributed to the spread of the epidemic (Rabobank, 2020).

Unique aspects of the Chinese food economy also contributed to the spread of ASF. The Chinese pork market is largely non-automated and dependent upon “warm meat” processing (Figure 1). This system of slaughter and transport relies on truck-based refrigeration that delivers pork to markets within 24 h of slaughter (Standaert, 2020a), presenting challenges for disease outbreak tracing and containment. The food culture of China is based upon “wet market” distribution, meaning that fresh meat (usually domestic species, but sometimes including wildlife), seafood, and produce are offered at a specific location. Though the terms are often used interchangeably, wet markers differ from “wildlife markets,” which specialize in the sale of live farmed and wild-caught wildlife (Warchol, 2004). Wet markets complicate ASF control as live pigs and pig products may be closely associated at these sites (Standaert, 2020a).

Stability of the Chinese pork market faltered as ASF decreased pork supply. Government actors and suppliers looked to other protein sources to meet demand, which rapidly induced global impacts on other commodity markets (Rabobank, 2020). The demand for alternative protein sources may have also impacted wildlife markets and production systems. The Chinese government historically encouraged wildlife trade as a form of rural economic development, enhancing through policy rather than investment both farmed wildlife production and wild harvest (Standaert, 2020b). Given its unofficial status, this sector is prone to poor regulation, and official statistics on pricing or production are scarce. While it is unclear how disruption to pork markets may have affected activity at wildlife markets, the convergence of circumstances outlined here, with first the incursion of ASF virus into China followed closely with the first detection of SARS-CoV-2 virus, suggest that acceleration of COVID-19 cases due to severe disruption of the Chinese pork market is plausible, possibly through increased congregation of vendors and consumers at the markets.

The ASF outbreak illustrates the broad need for the use a One Health approach in disease management in that a convergence of animal, human, and environmental conditions resulted in its ignition and subsequent epidemic spread (Figure 1). Addressing the consequences of the outbreak relating to food insecurity and potential indirect amplification of SARS-CoV-2 emergence and the continuing spread of ASF across the globe will require a focused effort among basic scientists, epidemiologists, industry, and governmental representatives. Convergence of specific technologies in vaccine development in both the veterinary and human medical fields, for example, could be a solution to the control of both diseases (Vrba et al., 2020).

## COVID-19

During the ASF outbreak in Chinese swine markets, a cluster of pneumonia cases were reported in Wuhan city, Hubei province, China, starting in late 2019, and reported to the World Health Organization (WHO) on December 31, 2019 (Wu et al., 2020). Initial cases were linked to Huanan Seafood Wholesale Market, causing public health officials to suspect a zoonotic origin.

Throughout the early stages of the pandemic, there was a great degree of speculation as to the evolutionary origins of SARS-CoV-2 and the animal species involved in the spillover event to humans (Zhang and Holmes, 2020; Good, 2021). Virologists and epidemiologists conducted extensive environmental and animal sampling at the Huanan seafood market to determine whether SARS-CoV-2 was present at the Huanan market in December 2019. In May 2020, the director of the Chinese Centers for Disease Control and Prevention announced that all animal samples tested for SARS-CoV-2 were negative, suggesting that the Huanan Seafood Wholesale Market was likely a point-source outbreak sourcing additional human-to-human transmission chains rather than the location where the initial animal-to-human transmission event took place. On February 24, 2020, the Chinese government instituted a ban on the trade and consumption of non-aquatic wildlife modeled on prohibitions instituted after the 2003 SARS-CoV outbreak, linked to trade in civet cats, that had since been relaxed in the face of mounting social and economic pressures (Mitchell et al., 2020). The WHO-convened global study of origins of SARS-CoV-2: China Part evaluated several possible wildlife sources for the virus, ultimately drawing no significant conclusions (WHO, 2021). Additional studies are ongoing to continue to unravel the details of the origins of SARS-CoV-2.

The spillover event that initiated the COVID-19 pandemic extended well beyond the boundaries of wet markets, moving around the globe in a way that altered the fabric of society. The scope of economic, political, and social consequences of the COVID-19 pandemic is engrained in public consciousness. However, the comparison and interdependence of the ASF outbreak and COVID-19 pandemic, has not been widely evaluated, particularly under the lens of defining One Health solutions.

## Discussion: Comparison of Parallel Pandemics

African Swine Fever and COVID-19 are strong examples of the need for applying a One Health approach to disease control as they demonstrate the devastating results of contagious spread of a virulent infection across a significant portion of the globe owing to animal, human, and environmental interactions. Prediction, prevention, mitigation, and restoration phases of such outbreaks require consideration of cultural, political, industrial, economic, nutritional, and psychological components of complex, overlapping societies and habitats. It is impossible to “solve” One Health pandemics unilaterally. Instead, the effort required to manage a pandemic requires resilience, unity, and foresight.

We have outlined striking similarities between the complex biological histories and complicating factors that resulted in rapid spread and stymied mitigation efforts in the coincident ASF and COVID-19 pandemics. Both viruses are multi-host pathogens, complicating our understanding of the origins and/or epidemiology of the virus within larger-scale systems. Both viruses have a suspected connection to wildlife disease spillover. ASF is enzootic in many wild boar populations at a prevalence high enough to facilitate periodic spillover into domestic swine populations, while SARS-CoV-2 is speculated to have its origins in *Rhinolophus* spp. Bats (Andersen et al., 2020; BBC News, 2020).

Both pandemics highlight the difficulties in adequately preparing for and containing an outbreak in the face of complicating social and political factors. China published an ASF contingency plan in 2015 requiring the culling of all pigs within a 3 km radius of the initial site (Wang et al., 2019). However, initiation of this plan resulted in stigmatization of reporting and adversely impacted compliance (Patton, 2020). Governmental subsidies were inadequate to support farmers with culled herds, and enforcement of transport and slaughter regulations was sometimes poor (Patton, 2020). Aggressive testing and contact tracing were critical to the early containment of COVID-19, as reflected by the discrepancy in outcomes in different regions. For example, Vietnam, Singapore, and Taiwan – having been significantly affected by the 2003 SARS outbreak and avian influenza – had developed infrastructure to deal with a highly transmissible respiratory pathogen (Institute of Medicine, 2004). As a result, these countries witnessed lower fatality rates than the United States, Italy, and other countries that implemented less aggressive diagnostic protocols and social distancing measures (Villani et al., 2020). As the second and third waves of the virus have crested, rapid detection and quarantine have been repeatedly shown as the most effective measures of control (Lotfi et al., 2020).

Controversies have arisen over implementation of control measures for human-to-human transmission of SARS-CoV-2 in the United States including quarantine time, mask-wearing, and social distancing. Similarly, there has been debate about eliminating backyard pig production systems to prevent ASF, as these operations lack appropriate biosecurity measures and are therefore a risk factor for swine epidemics and zoonotic disease emergence and spread. However, because these systems support the welfare and livelihoods of smallholder farmers, their loss would negatively impact resource-restricted communities (Huang et al., 2012). Personal freedom, mental health issues, and economic concerns are all cited as reasons to decrease protective regulations even in the face of active disease spread. Under-reporting of disease incidence and misinformation about risk factors could have contributed to the rapid growth of COVID-19 outbreaks in the United States and other countries, indicating that the challenges noted in China’s official response to both ASF and COVID-19 also occurred in other countries with different governing systems (Patton, 2020; Ruinyan et al., 2020).

The ASF and COVID-19 pandemics likely had asymmetric effects on each other, many of which are difficult to quantify. As the ASF epidemic preceded COVID-19, it likely influenced the spread and severity of the burgeoning COVID-19 pandemic. As previously noted, pork shortages possibly drove dietary changes to other protein sources, potentially increasing human-to-human contact and reliance on wildlife farming that may have exposed individuals to reservoir or intermediate hosts for SARS-CoV-2 (Woonwong et al., 2020). Slaughterhouses were also found to be the location of point source outbreaks, both in China and around the world (Taylor et al., 2020). The influence of COVID-19 on the spread of ASF is not as significant but still conceivable. The pork processing industry in China is highly reliant on manual labor (Pan et al., 2020). The spread of COVID-19 sharply limited the availability of the labor force at a time when the inspection, testing, and culling of pigs demanded an increase. In addition, imports of meat in the 2019–2020 winter were unable to be promptly transported from Chinese ports due to COVID-19 transportation disruptions and labor shortages (Chandran, 2020). The impacts of the compounded economic, dietary, and psychological stressors caused by the two pandemics are difficult to quantify, and the indirect and downstream effects of the pandemics on both provisioning of public health resources and exacerbation of health inequities will be delineated by public health agencies worldwide for years to come.

The ASFV and SARS-CoV-2 outbreaks illustrate the importance of having scalable vaccination technologies available. Due to lack of an effective vaccine, ASF is extremely difficult to control or prevent; therefore, outbreak control relies on drastic measures like culling infected animals. Although highly impactful, the non-zoonotic ASFV lacked the political urgency of SARS-CoV-2. The development of a COVID-19 vaccine relied on years of coronavirus and vaccine efficacy research spurred by SARS-CoV and MERS-CoV outbreaks, unprecedented funding from private industry and government, and an acceleration of regulatory process (Ball, 2020).

Finally, genetic mutations have arisen in both SARS-CoV-2 and ASFV owing to evolutionary processes that occur when a virus circulates through large swaths of naïve populations (Chen et al., 2020; Lauring and Hodcroft, 2021). The advent of more transmissible SARS-CoV-2 variants has complicated control efforts globally. Similarly, ASFV has lingered in parts of Hong Kong and mainland China because a new variant resulting in milder disease makes detection far more difficult. While this variant is associated with decreased mortality in swine, infection results in increased abortion and long-term unthriftiness, both of which have devastating impacts on the industry (Sánchez-Cordón et al., 2018; Friends of the Global Fight, 2020).

The ASF and COVID-19 outbreaks have demonstrated that non-pharmaceutical interventions – such as quarantine and stamping out – have the most impact on whether an outbreak was contained. It has been shown that ASFV likely spread from Georgia (isolates identified in 2007) through Russia and then through wild swine into China (Rowlands et al., 2008). If strict control measures had been implemented along that chain, the eventual epidemic in China could have been minimized. The economic benefits of those initial measures are difficult to quantify, but the concept of them is not. Similarly, the initial COVID-19 outbreak expanded quickly due to weak quarantine protocols and movement restrictions. The delay to instigate these measures – amplified by delayed reporting and misinformation – led to the global pandemic we are experiencing.

## Downstream Consequences of COVID-19 and ASF

There are many consequences of the both COVID-19 and ASF outbreaks, including economic and social upheavals (Supplementary Table S1). A better understanding of these consequences could aid in risk reduction of future scenarios, promoting positive outcomes and resiliency. Additional emerging infectious disease outbreaks are a significant concern, as medical and diagnostic supply infrastructure is currently stressed by urgent needs of these two pandemics. In the United States, many national animal health and veterinary diagnostic laboratories assisted with SARS-CoV-2 diagnosis, limiting capacity to surveil for ongoing zoonotic and endemic diseases of animals. Changes in human behavior during the pandemic have resulted in record numbers of salmonella outbreaks (from backyard chicken rearing) and a fear of increased cases of Lyme disease (attributed to increased outdoor activities in the midst of climate patterns favoring tick populations) as well as increased risk of health consequences due to inactivity, weight gain, and mental health issues (Centers for Disease Control, 2020b; Mock, 2020). Additionally, increased death rates have been noted and are suspected to be due to “medical distancing” secondary to restricted access to health care and/or fear of SARS-CoV-2 infection at health care facilities (Mihaljevic and Farrugia, 2020).

Civil and social unrest, permanent modification of workplace and educational frameworks, and changes in protein consumption patterns are likely to be key outcomes of these two pandemics. On the positive side, investments to advance diagnostics, therapeutics, vaccines, and other solutions for infectious disease mitigation are rapidly developing, likely with impact far beyond COVID-19 and ASF (Figure 1).

## How Do We Prepare a One Health Approach for the Next Pandemic?

Why has it been so hard to prepare for pandemics that have been repeatedly documented as a threat to the lives of millions of animals and humans? And, what can we do to reverse this predictable trend?

Social, cultural, and political factors underlie our seeming inability to prepare for disease outbreaks. Pandemics are exceedingly rare events relative to the number of human-animal-environmental interactions. For example, primary factors leading to SARS-CoV-2 emergence (human-animal interactions at wild-urban interface) and ASFV spread (transport of food products across international borders) are events that happen routinely around the world. Low-risk, high-impact events resulting in infection in a target population cause outbreaks that do not usually amount to epidemics. However, the frequency of pandemics will likely increase in the future. There have been at least three zoonotic coronaviruses in the last two decades. Potent drivers like globalization, expanding population, and pressure on the human-wildlife interface will increase this likelihood. Investment in detection of human illness (versus preventing the myriad of interactions with exceedingly small probabilities of ignition) would overcome the need to eliminate practices and behaviors that are vital to community identity. Development of strong local and regional surveillance networks, with incentivized data sharing and open communications, is essential to change outcomes of future spillover events.

**FIGURE 2:**
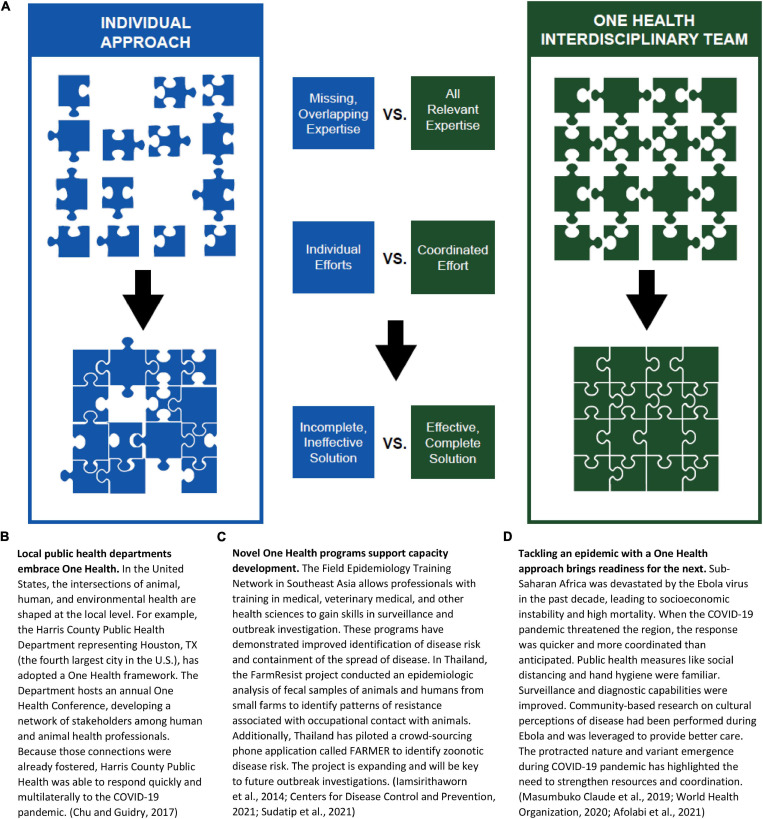
A One Health Approach: Framework and Vignettes. **(A)** A One Health approach brings together a diverse, inclusive, multidisciplinary team of experts to address complex problems resulting in coordinated, effective, complete solutions. One Health teams incorporate individuals from all science disciplines including but not limited to data, math, computer, engineering, behavioral, social, economic, cultural, natural, applied, biomedical, agricultural, and environmental sciences. Creating a framework for One Health teams with international connections can decrease the challenges and costs associated with multiple individual efforts. **(B–D)** Vignettes representing a One Health Approach.

Successful pandemic preparedness, however, must expand beyond local and regional borders. Disease is not constrained by boundaries of country or category. ASFV may not be zoonotic, but it has far-reaching impacts on the human population that involve economics, nutrition, environmental management, trade, food security, wildlife interactions, etc. Similarly, the impact of SARS-CoV-2 is far from just a human health concern and has affected nearly all aspects of human life around the globe–including airline travel, consumption trends, environmental impacts, and mental health. The multifaceted impacts of the ASF and COVID-19 pandemics strongly support the need for a One Health approach to pandemic management that incorporates a team of diverse and transdisciplinary experts to cooperatively solve complex problems (Figure 2).

One Health requires an inclusive process that breaks down barriers and brings together professions. Creating a funded network of One Health teams and Centers of Excellence across the United States that link to other networks such as those in Africa (African One Health University Network) and Southeast Asia (Southeast Asia One Health University Network) would provide a stronger, more coordinated means of addressing worldwide problems. Areas for investment to influence early phases of pandemics include: (1) enhancement of local surveillance efforts, with capacity for data storage and analysis to detect new infections; (2) communication strategies at local, regional, national, and global scales, incentivized by investment of resources and recognition of scientific expertise and public health management; (3) international training programs that inspire disciplinarily diverse early-career scientists collaborate; and (4) One Health legislation and investment to operationalize roadmaps that outline plans for mitigation of future pandemics. Understanding and mobilizing a One Health framework allows for the lessons and structures from one outbreak to be utilized and improved for the next one, regardless of whether the disease impacts humans or animals.

A One Health approach has immense potential to improve future outcomes not only for infectious diseases but other shared problems too. Creating a One Health framework that facilitates finding solutions to the “other shared problems” may be the key to truly successful disease outcomes. As Peter J. Hotez, a physician and vaccine developer, is quoted saying, “We must remove the conditions in which new diseases arise: poverty has more impact than any of our technical interventions…. Political collapse, climate change, urbanization, deforestation: these are what’s holding us back. We can develop all the vaccines and drugs we want, but unless we figure out a way to deal with these other issues, we’ll always be behind” (Mckenna, 2020).

## Data Availability Statement

The original contributions presented in the study are included in the article/Supplementary Material, further inquiries can be directed to the corresponding author/s.

## Author Contributions

SV, CT, AF, TW, and GW wrote the manuscript. SV and TW conceived of the manuscript idea and discussion. GW contributed on content about wildlife trade. AF researched and wrote about COVID-19 and designed Supplementary Figure S1. CT researched and wrote about African Swine Fever, designed Figure 1, and prepared the manuscript. TW designed Figure 2. EA reviewed the manuscript and contributed to discussion of implications. All authors contributed extensively to the work presented in this manuscript.

## Conflict of Interest

The authors declare that the research was conducted in the absence of any commercial or financial relationships that could be construed as a potential conflict of interest.

## Publisher’s Note

All claims expressed in this article are solely those of the authors and do not necessarily represent those of their affiliated organizations, or those of the publisher, the editors and the reviewers. Any product that may be evaluated in this article, or claim that may be made by its manufacturer, is not guaranteed or endorsed by the publisher.
