# Supplementary material for: Parallel Pandemics Illustrate the Need for One Health Solutions
Source: Front Microbiol. 2021 Oct 8;12:718546. doi: 10.3389/fmicb.2021.718546 (PMC8532541; doi:10.3389/fmicb.2021.718546)
Supplement: Supplementary file 1 [file Data_Sheet_1.docx]

**Online Supplementation**


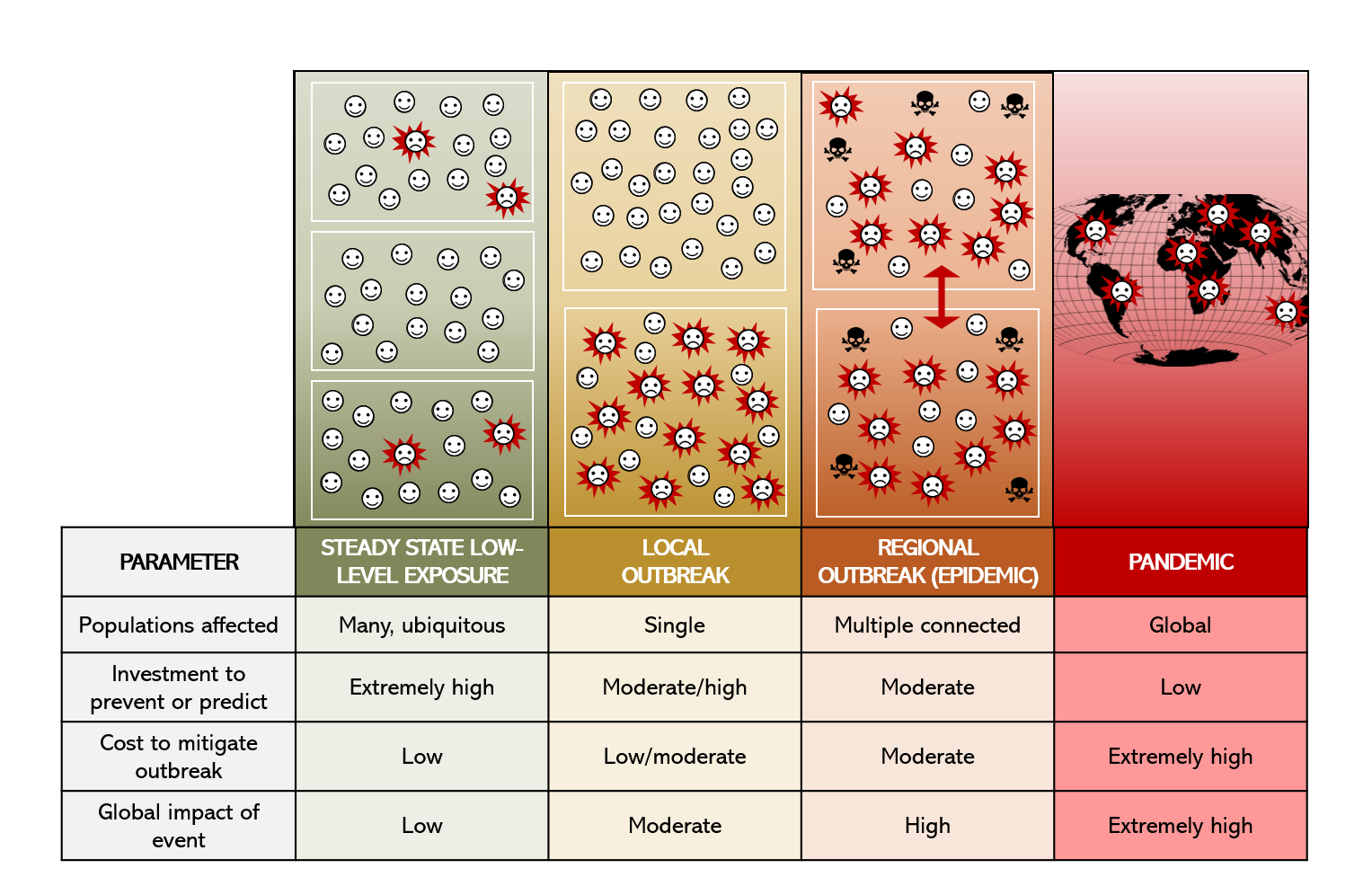


**Figure S1. One Health pandemics are launched by low-risk, massive-impact events.** Humans and animals are engaged in a constant level of ‘steady-state’ activities that could potentially result in pathogen transmission. Most of these situations do not result in competent infection (panel A). However, rare spillover events following pathogen-individual interactions results in an ‘index case’, illustrated in Panel B. Infection in one individual does not typically result in a pandemic, but local or regional infections might occur when pathogens are well-suited for infection of the new host (Panel C). Regional outbreaks can potentially spread globally through transportation networks or via efficient individual to individual spread (Panel D). The investment to prevent or predict spread is best deployed at the local or regional scale to focus on true outbreak settings before mitigation costs are extraordinarily high. Investment in infrastructure for early detection and incentivizing early reporting and mitigation would minimize the risk of global pandemics.

| Positive Direct/ Indirect Effects | Negative Direct/Indirect Effects |
| --- | --- |
| Acceleration of discoveries that allow more rapid and accurate disease diagnosis | Worsening of health and wealth disparities |
| New vaccine and therapeutic approaches and improved understanding of virus-host interactions | Worsening of food insecurity |
| Empowerment of a new generation of politically active citizen | Amplification of misinformation campaigns and distrust of government agencies |
| Decreased carbon emissions from significantly curtailed global travel | Increase in incidental diseases due to behavioral changes |
| Decreases in communicable diseases resulting from public health practices | Increase in secondary disease from health care disruption |
| Neutral Direct/Indirect Effects | |
| Changes in protein consumption patterns | |
| Permanent modification of workplace and educational practices | |
| Shifts in geopolitical power and economic structures | |

**Table S1. Downstream consequences of COVID-19 and ASF pandemics.**
